# The autism spectrum disorders (ASD)

**DOI:** 10.1186/1824-7288-40-S1-A67

**Published:** 2014-08-11

**Authors:** G Gambino

**Affiliations:** 1 Head of Surgery HUB 2nd level for Intensive Early Diagnosis and Treatment of Autistic Syndromes, UOC NPIA ASP Palermo, Italy

## 

Screening within the 2nd year of life ( primarily between 12 and 18 months ) is a tool for early detection of the risk of a neurobehavioral developmental disorder and is a chance to change the term prognosis of ASD.

The Analysis of the interactions of functional and dysfunctional interactive communicative signals from the very early ages of life, as well as of motor patterns , allows us to capture the significant elements of the neuro-behavioral development in children at risk of developmental ASD.

The ASD as the primary difficulties of social orientation and inter-subjectivity, which leads to not meeting the needs of dyadic relationship becomes even cognitive deficits, given that the abnormal behavior and the failure to derive anomalies early experiences of the process of neuronal growth.

The diagnostic label of ASD in the transition from DSM-IV TR DSM 5 : basically goes from one classification categorical one dimensional paintings that defined and distinct switching to the concept of spectrum as a continuum of clinical features (anomalies qualitative different degree of movement and the axis linguistic communicative) until the extension to the poles towards frameworks neurodiversity.

The spectrum autism disorders in relation to the clinical expression of the core autism ( social and communicative disorders and restricted interests and repetitive, stereotyped ) occurs at three levels of severity , in line with the possibilities prognostic constitute the consideration for the needs and welfare of the degree of complexity in the "care" the person affected by ASD and his family.

There is no genetic marker of autism but there is a multi- gene and environmental control of the clinical expression of the responsible individual developmental trajectories of endophenotypes.

Developments in neuroscience constitute the antecedent of the models of cognitive behavioral treatment oriented, validated for the effectiveness in accordance with the Guidelines ISS. If activated at an early stage, it is more effective than subsequent treatments, resulting in significant improvements in language, IQ, and adaptive behaviors.

